# Strength, Microstructural, and Environmental Performance of Phosphogypsum–GGBS–Carbide Slag Ternary Blended Cement

**DOI:** 10.3390/ma18214953

**Published:** 2025-10-30

**Authors:** Yunzhi Tan, Joseph Roland Atenga Essama, Chong Wang, Jun Wu, Huajun Ming

**Affiliations:** 1 Yichang Key Laboratory of the Resources Utilization for Problematic Soils, China Three Gorges University, Yichang 443002, China; yztan@ctgu.edu.cn (Y.T.); essarold@yahoo.fr (J.R.A.E.); wujun@ctgu.edu.cn (J.W.); hjming@ctgu.edu.cn (H.M.); 2 Institute of Problematic Soils Mechanics, China Three Gorges University, Yichang 443002, China

**Keywords:** carbide slag, ternary cement blended, strength, eco-binder, synergistic activation

## Abstract

Phosphogypsum (PG) and Carbide Slag (CS) are two industrial byproducts that can be used as cementitious materials, and their synergistic effect provides excellent activation of ground granulated blast furnace slag (GGBS), which is traditionally activated by lime (LM). However, the behavior of PG-GGBS-CS ternary blended cement remains largely unexplored. In this study, the mechanical performance, hydration mechanisms, and environmental profile of PG–GGBS–CS binders in comparison with PG–GGBS–LM were evaluated by unconfined compressive strength (UCS), XRD, SEM, and TGA analyses. The optimum formulation containing 30% PG achieved 24.88 ± 1.24 MPa at 28 d, statistically comparable to 25.6 ± 1.28 MPa for PG–GGBS–LM. The synergistic activation of PG and CS/LM on GGBS has been identified as a crucial factor in the strengthening of UCS in ternary blended cement. Hydration products consisted mainly of calcium silicate hydrate (C–S–H) gels and Ettringite (AFt). Importantly, the CO_2_ footprint of PG–GGBS–CS was reduced by 3.2% compared to that of PG–GGBS–LM. These findings establish CS as an effective substitute for lime in eco-binders, combining technical efficiency with carbon mitigation and offering a viable pathway for large-scale valorization of hazardous industrial residues.

## 1. Introduction

Phosphogypsum (PG) is a hazardous waste from the phosphoric acid (H_3_PO_4_) industry [1,2,3]. Each ton of phosphoric acid produces around 5 tons of PG, which contributes to an estimated annual production of 100–280 million tons of PG worldwide [4,5,6]. Ground granulated blast furnace slag (GGBS) is a byproduct of ironmaking, which is regarded as an excellent substitute for cement in construction [7]. The production of 1 ton of GGBS results in the emission of 0.07 tons of carbon dioxide (CO_2_), which is much less than that of Portland cement, approximately 0.95 tons [8,9,10]. These wastes have significant environmental and economic impacts, with the potential to cause harm if not properly managed.

Interestingly, GGBS combined with a small amount of alkaline activator (such as lime or cement) can produce an environmentally friendly ternary blended cement that replaces Portland cement, which is crucial for sustainable construction practices [11,12]. Recently, many researchers have investigated the activating effect of lime and cement on this ternary blended cement. S Wild et al. [13] and Li and Zhang [14] have demonstrated that the utilization of GGBS-LM blended cement results in a strength enhancement of the soils in the construction of motorways and foundations. The research conducted by Bolang Z et al. [15] has demonstrated that the combination of GGBS and cement results in the production of high-performance concrete structures with satisfactory strength. Additionally, the study conducted by Tan et al. [16] has shown that the combination of GGBS and cement increases the unconfined compressive strength (UCS) and promotes the formation of a more compact cementitious matrix.

PG can further improve the activation effect of GGBS with LM or OPC by the formation of Ettringite, which can be interspersed between calcium sulfate dihydrate crystals to optimize the pore structure. However, few studies have been found in terms of the behavior of PG-GGBS-LM ternary blended cement. The studies conducted by Huahui Qi [17] indicate that the combination of GGBS and PG enhances the compressive strength of cementitious composites. Similarly, Pratap [18] revealed that GGBS combined with PG improves the microstructural properties and strength of concrete by making it denser and more compact. Gutsalenko [19] and Kai Gu [20] have shown that GGBS can be activated by PG, improving its reactivity and mechanical properties. This activation leads to increased UCS and durability in concrete applications and promotes the formation of beneficial hydration products that improve the overall performance of the material in construction. Nicula [21] revealed that PG activation of GGBS exhibits improved long-term durability properties and a UCS that increases with curing time, due to the formation of stable hydration products such as C-S-H and Ettringite (AFt). Based on the above, cement or LM combined with PG is a viable alternative to co-activate GGBS and stimulate a significant influence on the mechanical properties of alkali-activated materials (AAMs) derived from industrial byproduct composites. However, cement and lime are both materials with high carbon emissions. Therefore, identifying a low-carbon activator that can replace lime or cement without compromising performance is essential.

Carbide Slag (CS), an off-white industrial waste, has the potential to partially or fully replace lime and to be used as an alkaline activator for the activation of GGBS as a building material [22]. In 2020, global CS production was approximately 45 million tons [23], with China accounting for over 36 million tons, and total CO_2_ emissions due to the production of CS exceeded 5.6 million tons [24]. Furthermore, the co-activation of PG/CS or PG/LM to activate GGBS improves soil stabilization [25], makes the cementitious material denser and more compact, and thus promotes excellent mechanical performance, making CS a viable substitute for LM [22,26]. In addition, when CS is replaced by LM, the solidified cementitious material exhibits a reduced carbon footprint [27,28]. However, the activation behavior of CS in PG–GGBS ternary blended cement remains unexplored, representing a crucial research gap.

The objective of this work is to evaluate whether CS can effectively replace lime as an activator in PG–GGBS ternary binders while maintaining comparable strength and hydration characteristics. The influence of CS and PG content, as well as curing time, on the mechanical, microstructural, and environmental performance of PG–GGBS-CS ternary blended cement is systematically assessed using UCS, XRD, SEM, and TGA. In addition, these properties are also compared with those of PG–GGBS-LM, resulting in sustainable, low-carbon binders. It is anticipated that these findings will constitute a substantial advancement in the utilization of eco-binders and their applications, with the objective of reducing environmental impact and promoting more economical and sustainable construction practices.

## 2. Materials and Methods

### 2.1. Materials

The principal materials used in this study were Phosphogypsum (PG), ground granulated blast furnace slag (GGBS), and Carbide Slag (CS). Table 1 provides a summary of the chemical composition of the raw materials. The gray-black PG was obtained from the Zhongfu Chemical Group Co., Yichang, China. The primary components of the PG are sulfate oxide (SO_3_), calcium oxide (CaO), and silicon oxide (SiO_2_), with a slightly acidic pH (6.4). The off-white GGBS was produced by Hubei Changyao New Material Co., Ltd, in Yichang, China. The primary constituents of GGBS are calcium oxide (CaO), silicon oxide (SiO_2_), and aluminum oxide (Al_2_O_3_), with a slightly alkaline pH (10.94). The CS was supplied by Chongqing Conch Cement Co., Ltd., Chongqing, China, and is primarily composed of calcium oxide (CaO), with a high alkaline pH (12.42).

A comparative analysis of PG, GGBS, and CS reveals that CaO is present in higher quantities of CS and in lower quantities of GGBS and PG, due to the presence of calcium hydroxide (Ca(OH)_2_), which is the main component of CS [29]. The proportion of SiO_2_ and Al_2_O_3_ is highest in GGBS, compared with those of PG and CS, which are responsible for the increased mechanical strength and excellent hardening durability properties of ternary blended cement.

**Figure 1:**
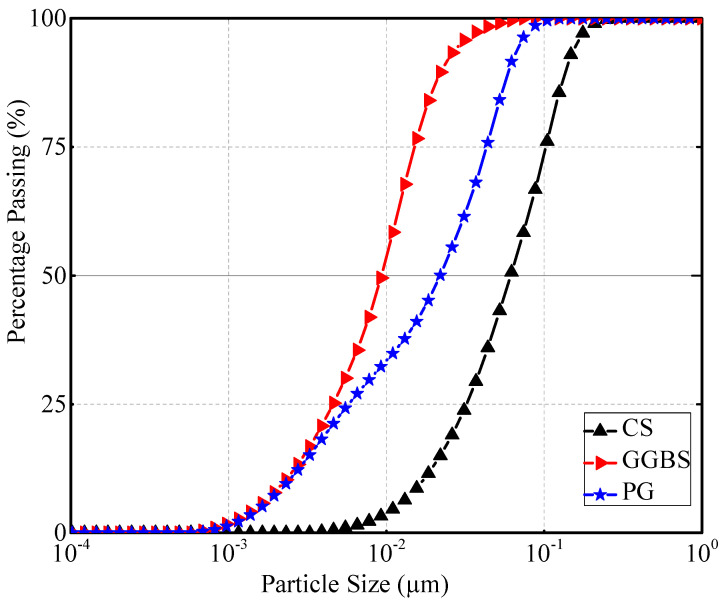
Particle size distribution curves of PG, CS, and GGBS.

**Figure 2:**
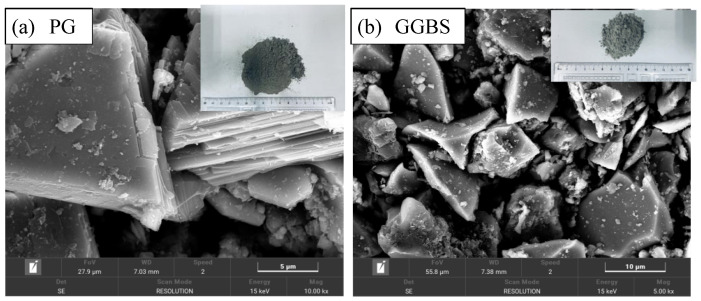
SEM images of PG and GGBS [25].

The particle size distributions and specific surface areas of the experimental materials, as illustrated in Figure 1, Table 1 and Table 2, clearly indicate that GGBS exhibits the finest particle characteristics, followed by PG, whereas CS is a comparatively coarse material. Similar findings have been reported by other researchers [14,30]. Figure 2 shows SEM images of PG and GGBS.

### 2.2. Sample Paste Preparation

The methodology for preparing ternary blended cement (PG-GGBS-CS) in the laboratory was conducted in accordance with Chinese standards [31]. The mixing ratios and material consumption per m^3^ of paste for PG, GGBS, and CS are presented in Table 3 and Table 4. First, the PG, GGBS, and CS materials were sun-dried for 24 h. The required quantities of PG, GGBS, and CS were weighed and combined in a Wanxiang UJZ15 mixer for approximately 45 s [32]. Next, ionized water (water/binder = 0.31) was introduced into the mixer, and a further 5 min of mixing was conducted to prepare the fresh, homogeneous ternary blended cement, which was then poured into 40 mm × 40 mm × 40 mm triple cylindrical molds and placed on a vibrating table for 5 min. Thereafter, the samples were covered with plastic film to eliminate air pockets and improve sample homogeneity. Finally, the prepared samples were placed in a sealed plastic container where the relative humidity and temperature were maintained at 95 ± 3% and 20 ± 2 °C, respectively. After 2 d, the stabilized ternary blended cement samples were demolded, labeled, cured and tested at day 7 and day 28.

## 3. Methods

### 3.1. Unconfined Compressive Strength (UCS) Test

After the samples were cured at day 7 and day 28, UCS testing was conducted in accordance with ASTM D21671M [33]. The automated EXCEED Model E45 testing machine (MTS Instrument Corp., Eden Prairie, MN, USA) coupled to a microcomputer was used to perform the tests at a constant displacement speed of 1.0 mm/min until the strain reached 15%. Three samples of each mixture were measured to obtain an average value.

### 3.2. Microstructural Characterizations

After UCS tests, the ternary blended cement samples cured at day 7 and day 28 were crushed and broken, respectively, and then subjected to XRD and SEM tests in order to better understand the microstructure and phase evolution of the ternary blended cement.

For XRD tests, samples were taken from the central portion. They were then subjected to termination of hydration with anhydrous ethanol and subsequent drying at 50 °C for a minimum of 24 h until reaching a constant weight. Subsequently, the dried samples were ground and filtered through a sieve (<75 µm) for XRD analysis to ensure particle size homogeneity prior to characterization. XRD data were recorded using a German Bruker D8 ADVANCE X-ray diffractometer (Bruker AXS GmbH, Karlsruhe, Germany).

For the purposes of SEM analysis, the samples were fragmented into blocks with a maximum dimension of 2 mm. Subsequently, the central sections were subjected to a five-minute heat treatment at 100 °C in a JFC-1600 furnace (Carbolite Gero Ltd., Hope Valley, UK) to terminate the hydration. Thereafter, the samples were fixed with conductive tape, and the surface to be observed was plated with gold [34]. Subsequently, alterations in the microstructure of hydrated ternary cement were examined using a Zeiss Gemini SEM 300 microscope (Carl Zeiss Microscopy GmbH, Oberkochen, Germany) at a magnification of 2000*.

A DTG-DSC analysis was conducted on samples of ternary blended cement. Initially, a powder measuring 10 mg was extracted from the central section of the ternary blended cement samples. This powder was then placed in an open corundum crucible and subjected to testing under nitrogen conditions using a thermal gravimetric analyzer (NETZSCH-Geratebeau GmbH, STA 449 F5, Jupiter, Selb, Germany). The measurement procedure was defined as a heating interval from 30 °C to 1000 °C at a heating rate of 10 °C/min, as previously described by Xingrun Wang [35].

## 4. Results and Analysis

### 4.1. UCS Analysis

**Figure 3:**
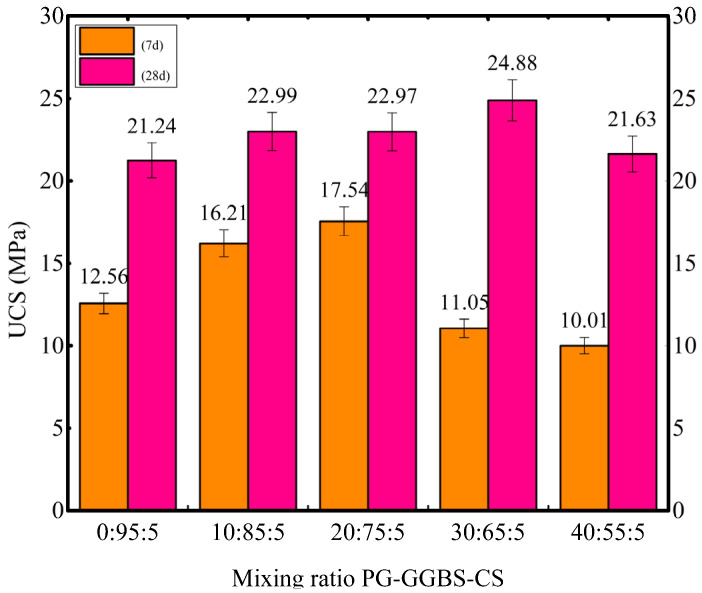
UCS of PG-GGBS-CS ternary blended cement at days 7 and 28.

Figure 3 shows the UCS results for PG-GGBS-CS ternary blended cement with PG contents ranging from 0% to 40% and cured at day 7 and day 28. It can be seen that UCS increases significantly with an increase in curing time, especially for the cement paste with a high PG content. For example, the UCS of cement paste with 20% PG increases from 17.54 ± 0.88 MPa at day 7 to 22.97 ± 1.15 MPa at day 28, increasing 30.96%. At the same time, the UCS with 40% PG increases by 116.08% from day 7 to day 28, which is much higher than that of the cement paste with 20% PG.

For the effect of PG content, it can be observed that the UCS of cement paste increases with PG content and then decreases, especially for short-term curing (7 d). At 7 d, UCS first increases with PG content (0–20%) from 12.56 ± 0.63 MPa to 17.54 ± 0.88 MPa and then decreases to 10.01 ± 0.50 MPa (PG content higher than 20%). The optimum PG content is 20%. However, with increasing curing time (day 28), the optimal PG content increases from 20% to 30%, whose optimum UCS value also increases from 17.54 ± 0.88 MPa at 20% PG content to 24.88 ± 1.20 MPa at 30% PG content.

PG primarily consists of CaSO_4_·2H_2_O (90–95%), with minor P_2_O_5_ (0.5–5%) [36,37]. The increased strength of PG-GGBS-CS ternary blended cement paste is primarily due to the hydration of GGBS, which is activated by CS and forms C-S-H/C-A-S-H gels. Then, sulfate in PG reacts with the formed C-A-S-H gels, and the Ettringite forms, further increasing the strength of the ternary blended cement paste. However, the P_2_O_5_ impurities in PG slow the hydration of GGBS and prolong setting time by forming phosphoric acid and hydroxyapatite (Ca_10_(PO_4_)_6_(OH)_2_), slowing the early hydration rate in PG-GGBS-CS blends as PG content increases. As a result, the optimum PG content increases from 20% (day 7) to 30% (day 28) after long-term curing.

### 4.2. XRD Analysis

**Figure 4:**
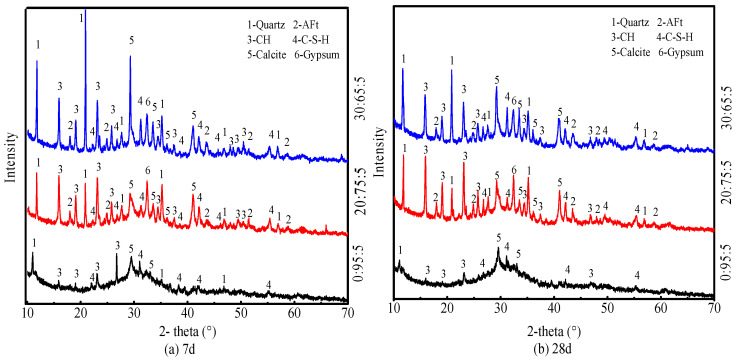
XRD patterns of PG-GGBS-CS ternary blended cement at days 7 and 28.

Figure 4 illustrates the XRD patterns of PG-GGBS-CS ternary blended cement with 0/20/30% PG content, cured at day 7 and day 28. The findings suggest that the primary minerals and hydration gels identified in the patterns are gypsum (CaSO_4_·2H_2_O), quartz (SiO_2_), Ettringite (AFt), portlandite (Ca(OH)_2_), calcite (CaCO_3_), and C-S-H gel [38]. However, in the ternary blended cement 0:95:5, no AFt was observed since no sulfate ions were provided.

The various peaks mentioned above are considered to be the result of the reactions arising below. The C-S-H peak is a consequence of GGBS hydration activated by CS and PG. Ettringite was formed as a result of the reaction between PG and GGBS [39]. The calcite peak is mainly attributed to the carbonation of CS during the curing period. The quartz peak is from non-hydrated GGBS and PG, as shown in Table 1. The portlandite (CH) peak is derived from the CS activator.

### 4.3. DTG-DSC Analysis

**Figure 5:**
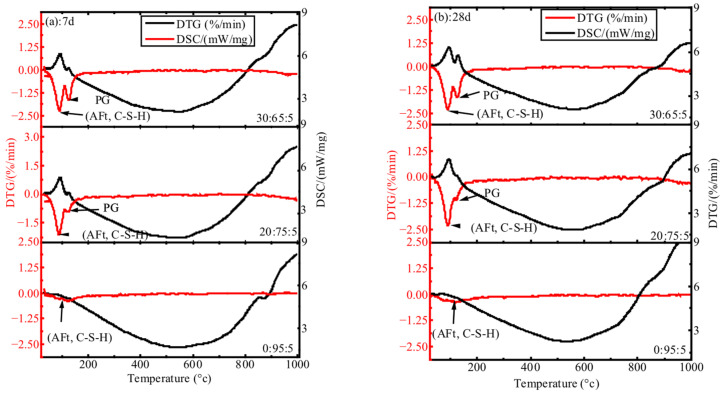
DTG-DSC curves for PG-GGBS-CS ternary blended cement at 7 and 28 d.

Figure 5 shows the DTG-DSC curves of ternary PG-GGBS-CS cement with 0/20/30% PG at hydration periods of 7 and 28 days. An intensified hydration phase is observed between 50 and 200 °C, marked by the presence of two peaks which improve as the PG content increases to 20 and 30% PG. The first peak results from the dehydration of the gels (AFt and C-S-H) [40,41], and the second from the decomposition of the PG [42]. The first peak is more developed with 30% PG, followed by the 20% PG peak, and tends to disappear at 0% PG content.

**Figure 6:**
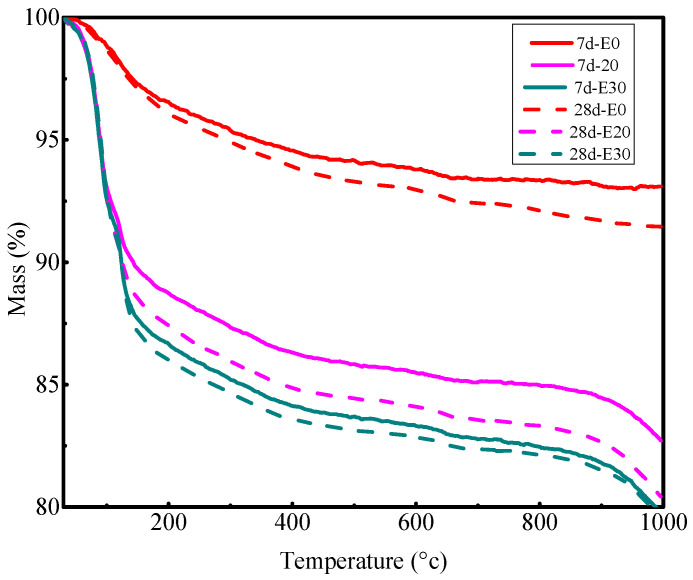
TDG-DSC curves for PG-GGBS-CS ternary blended cement at days 7 and 28.

Figure 6 illustrates the total mass loss of ternary blended cement PG-GGBS-CS at days 7 and 28 with 0/20/30% PG. It can be observed that the mass loss increases with curing time and the PG content. For example, the mass losses are 6.9%, 15.96% and 18.51% for 0/20/30% PG at day 7, respectively, increasing to 8.33%, 17.74% and 18.81% for 0/20/30% PG at day 28. It is clear that the co-activation effect of PG and CS on GGBS has a beneficial effect on the activation mechanisms of GGBS and on the evolution of the microstructure of the hardened ternary blended cement.

### 4.4. SEM Analysis

**Figure 7:**
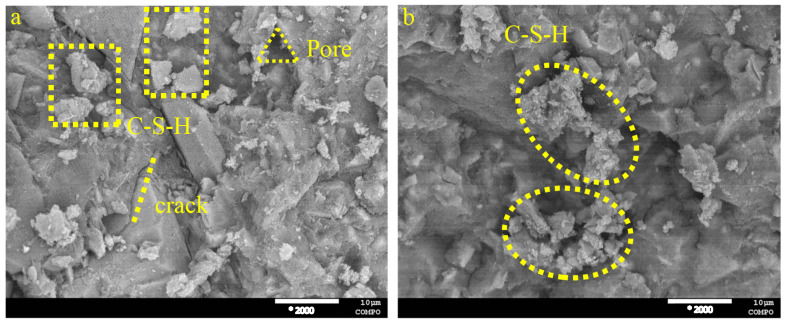
SEM images of PG-GGBS-CS ternary blended cement cured with 0% PG content: (**a**) day 7, and (**b**) day 28.

**Figure 8:**
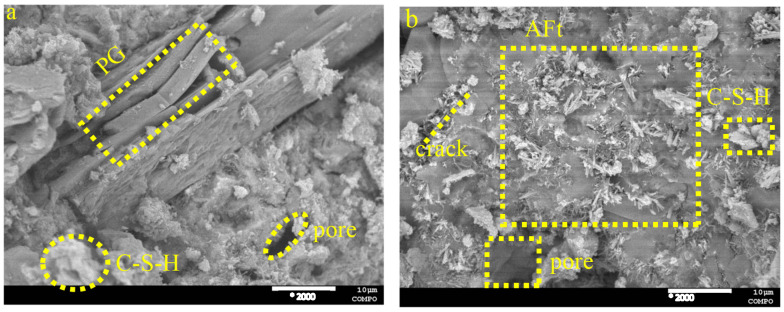
SEM images of PG-GGBS-CS ternary blended cement cured with 20% PG content: (**a**) day 7, and (**b**) day 28.

**Figure 9:**
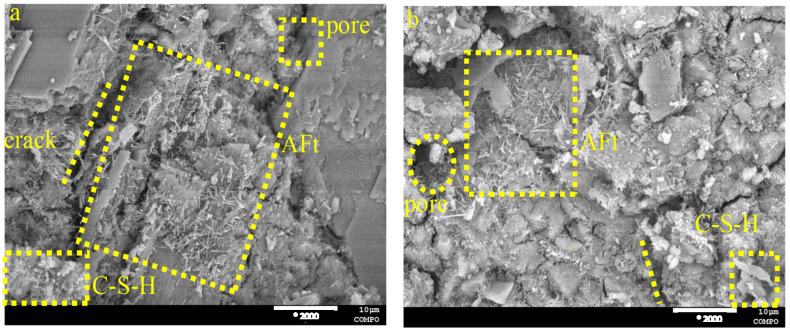
SEM images of PG-GGBS-CS ternary blended cement cured with 30% PG content: (**a**) day 7, and (**b**) day 28.

The hydration products were examined by scanning electron microscopy (SEM) at 2000* magnification to assess the microstructure and alterations of the hydration products in the cured paste PG-GGBS-CS ternary blend cement after 7 and 28 d, with PG contents of 0/20/30%, as shown in Figure 7, Figure 8 and Figure 9. The crystalline morphology indicates that the hydration products are mainly composed of C-S-H gels, in the form of clusters and needles AFt. Pores and cracks are also observed on the surface of the microstructure.

When the PG content is 0%, the formation of C-S-H gels, pores, and cracks is observed, as can be seen in Figure 7. However, when the PG content is 20 and 30%, significant needle AFt is observed in the ternary blended cement, and this increases with the PG content and curing time, improving the microstructure of the ternary blended cement, which becomes denser and more compact, as can be seen in Figure 8 and Figure 9.

In particular, the SEM analysis demonstrates the progressive microstructural evolution of PG-GGBS-CS ternary cement with varying PG content and curing time. At 0% PG, the hydration products primarily consist of C-S-H gels, forming a porous structure with visible cracks. When PG content increases to 20–30%, significant needle-shaped AFt crystals develop and become more abundant with both higher PG content and extended curing duration. This AFt formation, combined with C-S-H gels, progressively densifies and strengthens the cement matrix. The microstructural improvements correlate directly with the mechanical performance, showing that optimal PG incorporation (30%) creates a more compact and homogeneous structure through the synergistic formation of both C-S-H and AFt phases. These observations confirm PG dual functionality as both a sulfate source for AFt formation and a microstructural modifier in ternary cement systems.

## 5. Discussion

### Mechanical Behavior of PG-GGBS-CS and PG-GGBS-LM

**Figure 10:**
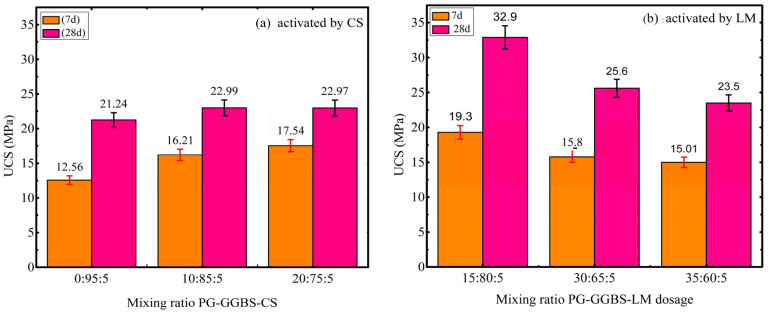
(**a**,**b**) Relationship between curing time and UCS of ternary blended cement.

A comparative analysis of PG-GGBS-CS and PG-GGBS-LM [43] ternary cements stabilized at 7 and 28 d at 0/20/30% and 15/30/35% PG content, respectively, is presented in Figure 10a,b, to compare the activating effect of CS and lime.

At day 7, the optimum UCS of PG-GGBS-LM is 19.3 ± 0.97 MPa with 15% PG content, while the optimum UCS of PG-GGBS-CS is 17.54 ± 0.63 MPa with 20% PG content. At day 28, the optimum UCS of PG-GGBS-LM is 32.9 ± 1.65 MPa with 15% PG, while the optimum UCS of PG-GGBS-CS is 24.88 ± 1.24 MPa with 30% PG. This shows that the UCS of ternary cement activated by LM is higher than that activated by CS, whatever the curing time, especially with low PG content. Furthermore, when PG is introduced at 30% on day 28, the UCS of PG-GGBS-LM and PG-GGBS-CS is almost similar.

This can be attributed to two main factors: the slightly higher CaO content in LM (97.07%) compared to that in CS (95.36%), as shown in Table 1, and the finer particle size of LM [42], which plays a crucial role in the co-activation of GGBS. Thus, the UCS of PG-GGBS-LM is higher than that of PG-GGBS-CS with low PG content. However, as the PG content increases, the activation of PG compensates for the deficiency of the low activating effect of CS, resulting in a comparable UCS between PG-GGBS-LM and PG-GGBS-CS.

Although PG contains impurities, no significant delay in hydration was observed in this study (Figure 10), suggesting that the synergistic activation with CS compensates for potential inhibitory effects.

Economic and environmental assessment of the PG-GGBS-CS and PG-GGBS-LM.

The material sustainability indicators (MSI) presented in Table 5, namely electricity consumption, CO_2_ emissions, and cost of materials, were used to assess and analyze the environmental and economic benefits of PG-GGBS-CS and PG-GGBS-LM [44,45]. The parameter MSI can be calculated as follows:

(1) $$MSI=\frac{UCS\;(\mathrm{MPa})}{Price\;(\mathrm{RMB}/t)\times CO_{2}\;(\mathrm{kg}/t)}$$

where *UCS* is the unconfined compressive strength (MPa) of the cement paste, *Price* is the price of ternary binder (RMB/t), and *CO*_2_ is the CO_2_ emissions of ternary binder (kg/t) during production, which are also summarized in Table 5, and can be calculated as follows:

(2) $${\mathrm{Price}}_{\mathrm{ternary}}=\Sigma (x_{i}\times C_{i})$$

(3) $$C=\Sigma (x_{i}\times C_{i})+C_{trans}$$

where x_i_ = mass fraction of component i, p_i_ = price of each component (Table 5), C_i_ = emission factor for component i, and C_trans_ = transport emissions (0.5 kg/ton-km).

The PG-GGBS-CS, PG-GGBS-LM, and PG-GGBS-OPC systems show superior sustainability performance compared to Portland cement. PG-GGBS-OPC provides the best strength/MSI balance (32.9 ± 1.65 MPa; 1.99 × 10^−5^), while PG-GGBS-CS stands out as the most cost-effective option (282.5 RMB/t) with a well-balanced profile combining mechanical strength (24.88 ± 1.24 MPa), low environmental impact (4.480 kg CO_2_/t), and economic efficiency. PG-GGBS-LM offers an intermediate alternative. Overall, these binders achieve 40–60% reductions in CO_2_ emissions and 30–50% lower costs compared to OPC, while maintaining adequate mechanical performance.

These findings are consistent with previous studies: James et al. [46] reported the cost and emission efficiency of lime-activated PG/fly ash binders, and Dang et al. [47] demonstrated the positive role of GGBS in strength and durability. Wu et al. [25] showed that the incorporation of the ternary stabilizer PG-GGBS-LM significantly improves the mechanical and environmental properties of dredged sludge and offers an economic advantage. However, the outcomes are sensitive to PG pretreatment (drying, calcination), the allocation method applied to GGBS (economic vs. mass-based), and transport distances. Moreover, long-term durability, particularly in terms of fluoride and phosphorus leaching, as well as moisture resistance, remains a critical area for future investigation.

**Table 5:** Economic and environmental cost assessment.

Specimens	Reference	Electricity Production (kWh/t)	28 d UCS (MPa)	CO_2_ Emissions (kg/t)	Cost (RMB/t)	MSI (×10^−5^)
PG	[48,49]	14.65		0.364	50	
GGBS		59.4		0.037	400	
CS		0		0.004	150	
LM		0.933		0.021	500	
Portland Cement		84.75	42.5	0.74	600	0.74
PG-GGBS-CS		38.61	24.88	4.480	282.5	1.96
PG-GGBS-LM		40.21	25.26	4.630	300	1.82
PG-GGBS-OPC		53.96	32.9	5.396	305	1.99

## 6. Conclusions

In this study, the co-activating effect of PG and CS on GGBS for the formulation of a PG-GGBS-CS eco-binder was investigated. A comprehensive experimental program, including UCS, XRF, XRD, TG/DTG, and SEM analyses, was conducted to evaluate the mechanical behavior, hydration mechanisms, and microstructural characteristics of PG-GGBS-CS ternary cements. The main conclusions are as follows:

(1) UCS of PG-GGBS-CS ternary cements shows a nonlinear evolution with PG content, peaking at 30% PG before declining. Without PG, ternary cements yield weak microstructures that do not form AFt. At 20–30% PG, sulfates from PG enable AFt formation while CS maintains alkalinity for C-S-H production, creating optimal densification. Beyond 30%, excess AFt may weaken the matrix. This highlights the critical balance between PG-CS and mechanical performance.
(2) XRD, TG-DTG, and SEM analyses revealed that hydration products increase with PG content (0–30%) after long-term curing, resulting in a denser microstructure. XRD confirmed the progressive formation of both crystalline (AFt, residual gypsum) and amorphous phases (C-S-H gels). TG-DTG highlighted the decomposition of hydrated phases (water loss from C-S-H/AFt) and gypsum (50–200 °C), with maximum hydration product mass at 30% PG. SEM showed reduced porosity and improved phase connectivity, explaining the densification.
(3) PG–GGBS–CS binders achieved mechanical performance statistically comparable to PG–GGBS–LM systems at higher PG contents, confirming that CS can effectively substitute lime while reducing carbon emissions by approximately 3.2%. This validates the research hypothesis that CS provides sufficient alkalinity and calcium to sustain efficient hydration and strength gain.

The findings demonstrate the technical feasibility and environmental benefits of CS-based activators in sustainable binder systems. However, this study was limited to compressive strength and standard curing conditions. Future work should evaluate flexural and tensile strength, durability under variable curing environments, and long-term performance to confirm field applicability. Overall, this study confirms that PG–GGBS–CS ternary cements offer a promising low-carbon alternative to conventional lime-activated systems, supporting waste valorization and circular economy strategies in sustainable construction.

## Figures and Tables

**Table 1:** Chemical composition of raw materials, in % by weight.

Chemical Composition	PG	GGBS	CS
SO_3_	49.88%	3.54%	0.30%
CaO	36.24%	37.21%	95.36%
SiO_2_	11.41%	38.26%	3.77%
K_2_O	1.03%	0.61%	-
Fe_2_O_3_	0.97%	2.02%	0.43%
Al_2_O_3_	-	16.42%	-
TiO_2_	0.17%	1.35%	-
L.O.I	0.30%	0.59%	0.14%
Initial moisture content	4%	0.96%	3.5%
Specific density (g/cm^3^)	2.6	2.8	2.4
Surface (m^2^/kg)	400	450	250
pH	6.4	10.94	12.42

**Table 2:** Particle size distribution of raw materials (D10, D50, and D90).

Diameters (µm)	PG	GGBS	CS
D10	5.67 µm	3.81 µm	16.94 µm
D50	52.23 µm	15.7 µm	61.32 µm
D90	141.9 µm	37.69 µm	137 µm

**Table 3:** Ternary blended cement ratio (%) by mass.

Serial	PG (%)	GGBS (%)	CS (%)	Water/Binder
0% PG	0	95	5	0.31
10% PG	10	85	5	0.31
20% PG	20	75	5	0.31
30% PG	30	65	5	0.31
40% PG	40	55	5	0.31

**Table 4:** Ternary blended cement consumption per m^3^ of paste.

Serial	PG (kg/m^3^)	GGBS (kg/m^3^)	CS (kg/m^3^)	Water (kg/m^3^)	Total (kg/m^3^)
0% PG	0	1417.7	74.6	462.6	1954.9
10% PG	148.6	1263.3	74.3	460.7	1946.9
20% PG	296.0	1110.1	74.0	458.8	1939.0
30% PG	442.2	958.2	73.7	457.0	1931.1
40% PG	587.3	807.5	73.4	455.1	1923.3

## Data Availability

The raw data supporting the conclusions of this article will be made available by the authors on request.

## Abbreviations

CS	Carbide Slag
GGBS	Ground granulated blast furnace slag
PG	Phosphogypsum
SEM	Scanning electron microscopy
UCS	Unconfined compressive strength
XRF	X-ray fluorescence spectroscopy
XRD	X-ray diffraction
TGA	Thermogravimetric analysis
AFt	Ettringite
CH	Portlandite
C-S-H	Calcium silicate hydrate
DTG-DSC	Derivative Thermogravimetry-Differential Scanning Calorimetry
PG-GGBS-LM	Phosphogypsum, ground granulated blast-furnace slag, and lime ternary stabilizer lime ternary stabilizer
PG-GGBS-CS	Phosphogypsum, ground granulated blast-furnace slag, and carbide slag ternary stabilizer
